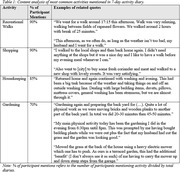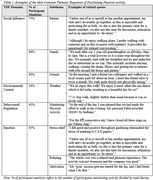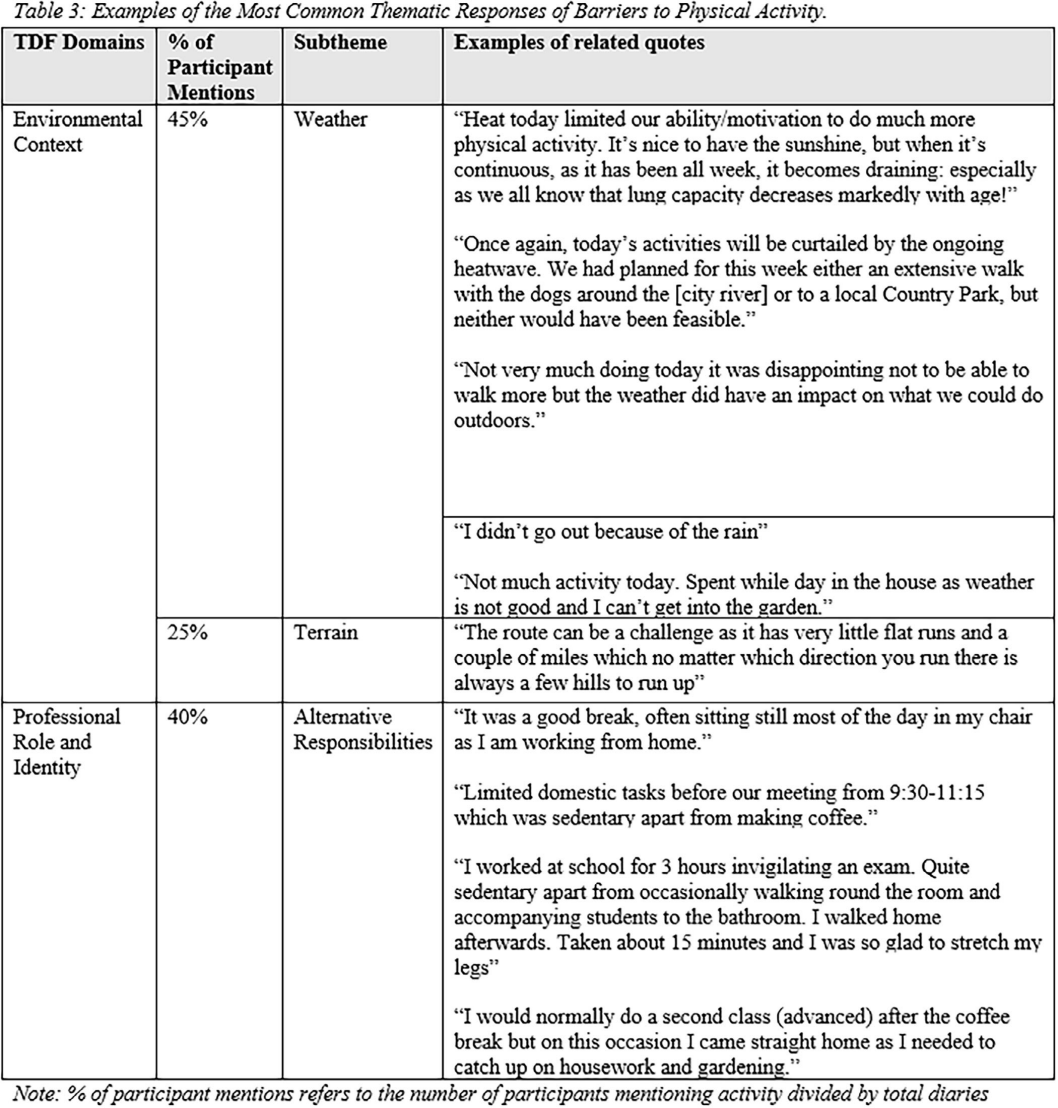# “My Wife and I Invariably Go Together”: A Mixed Methods Analysis of Physical Activity and its Influences on Older Adult Dyads

**DOI:** 10.1002/alz.089186

**Published:** 2025-01-09

**Authors:** Jenny L Wales, Calum Alexander Hamilton, Louis McCarthy, Sayeh Bayat, Gro Gujord Tangen, Neil Ireson, Vita Lanfranchi, Nicolas Farina, Ben Hicks, Ríona Mc Ardle

**Affiliations:** ^1^ Newcastle University, Newcastle‐Upon‐Tyne, Tyne and Wear United Kingdom; ^2^ Newcastle University, Newcastle upon Tyne United Kingdom; ^3^ Newcastle University, Newcastle Upon Tyne United Kingdom; ^4^ University of Calgary, Calgary, AB Canada; ^5^ Norwegian National Centre of Ageing and Health, Sem Norway; ^6^ University of Sheffield, Sheffield United Kingdom; ^7^ University of Plymouth, Plymouth United Kingdom; ^8^ Brighton and Sussex Medical School, Brighton United Kingdom

## Abstract

**Background:**

Physical activity (PA) is an imperative factor to healthy cognitive and functional ageing and may act as a protective factor against cognitive decline. Evidence suggests that as we age, PA declines, leaving a large proportion of older adults (OAs) ‘underactive’ and ‘unprotected’. Socialisation/social support is considered a beneficial influence on PA in OAs. It is unclear whether dyadic (e.g. spousal) influences impact PA in healthy ageing, and how this might impact digital activity monitoring or intervention development to support prevention of cognitive decline. This research aims to quantify volume and types of PA and explore influential factors for PA participation in OAs.

**Method:**

Participants were recruited via the ActivDyad study, where they wore a lumbar‐based‐accelerometer (AX6, Axivity) for 7 days, and completed a 7‐day activity diary. PA (i.e. daily steps) was derived from accelerometers using validated algorithms. Qualitative content analysis of the diaries was adopted for contextual understanding of the types and influences of PA. The Theoretical Domains Framework was utilised to identify common facilitators/barriers of PA in OAs.

**Result:**

21 dyads (42 OAs) participated in the study; 10 dyads were included in preliminary analysis (mean age (mean±SD): 68.6±4.8; 100% heterosexual & married; mean relationship time: 45±10 years; 80% retired). Participants carried out an average of 15,680 daily steps (range: 7,243‐22,668), and indicated their most frequent PA as: shopping, gardening, housekeeping, and recreational walks (Table 1). Participants identified potential facilitators (Table 2) and barriers (Table 3) for partaking in PA. Social influence (e.g. partner) was identified as most influential; 75% of participants mentioned this as a facilitator for PA. Environmental context, particularly weather conditions, were frequently discussed as both a facilitator (50%) and barrier (45%) to PA.

**Conclusion:**

Results suggest that dyadic interactions are a key facilitator of PA in OAs. Weather appears to have a bi‐conditional influence, promoting and/or discouraging PA; this may impact activity monitoring during adverse weather conditions (e.g. lower steps). Adherence may be higher in group PA interventions, and outdoor PA should be promoted in suitable weather. Analysis of the full sample will inform studies focusing activity monitoring and intervention development for dementia‐carer dyads.